# Optimization of off-grid renewable energy systems using a hybrid version of golden search algorithm

**DOI:** 10.1016/j.heliyon.2024.e30990

**Published:** 2024-05-14

**Authors:** Gengqiang Huang, Jie Gan, Ying Huang, Homayoun Ebrahimian

**Affiliations:** a Guangxi Technological College of Machinery and Electricity, Nanning, 530007, Guangxi, China; b Guangxi Economic and Trade Vocational Institute, Nanning, 530021, Guangxi, China; c Department of Engineering, Arak Branch, Islamic Azad University, Arak, Iran; d College of Technical Engineering, The Islamic University, Najaf, Iraq

**Keywords:** Off-grid electrification, Hybrid renewable energy system, Long-term capacity planning, Hybrid golden search algorithm (HGSA), Cost minimization

## Abstract

Currently, the development of HRESs (hybrid renewable energy systems) in remote areas is of great importance and popularity. However, measuring and optimizing the capacity of these systems faces a difficult challenge. Multiple works had been reported in the literature to optimize such systems, all of which aim to achieve an optimal configuration with minimum annual net cost. Therefore, the significance of providing off-grid electrification to remote areas through HRESs can be highlighted as a crucial case for sustainable growth. Accordingly, the study proposes a modified metaheuristic approach, known as the Hybrid Golden Search Algorithm (HGSA), for long-term application planning and optimization of the off-grid HRES. The aim of this algorithm is to minimize the amount of net cost which is used annually; to reduce the probability of power supply interruption. In order to assess the effectiveness of the proposed algorithm, a simulation study over a long period on a remote area was conducted. From the results, increasing the reliability level from 1 % to 3 % causes a decrease in the total net annual cost by around 7.3 % under the proposed HGSA and also results in a reduction in component size (around 6 % and 21 % reductions for the wind turbine area and storage tanks, respectively). Further, the HGSA technique obtains the lowest value of fitness function for the hybrid system at a reliability level of 3 %, which is 31,539,810$. This result demonstrates that the efficiency of HGSA outperforms Fuzzy Logic and Optimization, Artificial Bee Colony (ABC), and GSA techniques. Based on this, the proposed HGSA could lead to more promising results than the other comparative algorithms. Hence, the proposed HGSA can be a valuable tool for long-term application planning and optimization of off-grid HRES, which can contribute significantly to achieving sustainable development goals.

## Nomenclature

A_W_: Swept area
C: Cost ($)
C_p_: Power coefficient
E: Energy (kWh)
G_best_: Most optimum location
G_i_: i-th candidate in the solution space
I: Investment cost ($)
i: Interest rate
lb: Lower bound
n: Year number
N: Component numbers
NH_2_: H_2_ storage tanks quantity
P: Power (kW)
R: Replacement cost ($)
St: Maximum/minimum limitation
T: Function that decays over time
T_M_: Highest amount of iterations
ub: Upper bound
v: Wind speed (m/s)
$Z_{i}^{G}$: Global best position
$Z_{i}^{L}$: Current best position

**Greek symbols**
α: Additional weight to the PSO-based exploration
β: Reliability coefficient
β_D_: Reliability loss
η: Efficiency (%)
ρ_a_: Air density

**Subscripts**
ci: cut-in
co: cut-out
G: generation
Inv: inventor
L: load
r: rated

**Abbreviations**
ABC: Artificial Bee Colony
AC: Alternating Current
AI: Artificial Intelligence
DC: Direct Current
EL: Electrolyzer
ESS: Energy Storage System
ESSR: Enhanced Search Space Reduction
FC: Fuel Cell
GSO: Golden Search Optimizer
H_2_: Hydrogen
HGSA: Hybrid Golden Search Algorithm
HRES: Hybrid Renewable Energy System
HST: Hydrogen Storage Tank
LCOE: Levelized Cost Of Energy
LPS: Loss Of Power Supply
MVO: Multi-Verse Optimizer
OM: Operation And Maintenance
OP: Logic And Optimization
PIOA: Pigeon-Inspired Optimization Algorithm
PM-GWO: Position-Mutation Gray Wolf Optimization
POA: Pelican Optimization Algorithm
PSO: Particle Swarm Optimization
PV: Photovoltaic
SCA: Sine Cosine Algorithm
SSR: Search Space Reduction
STD: Standard Deviation
TNAC: Total Net Annual Cost
TSA: Tunicate Swarm Algorithm
WT: Wind Turbine

## 1 Introduction

energy distribution to remote places has been a major issue in recent years, particularly in developing nations where access to energy is restricted [1]. Off-grid electrification utilizing HRESs has received a lot of interest as a potential solution to this problem. HRES integrates some renewable energy sources including solar, wind, and hydro, for electricity generation that may be utilized to power homes, companies, and industries [2,3]. The usage of HRES can overcome the issues associated with delivering energy to rural places, such as high installation costs, restricted fuel access, and a lack of electricity networks [[4], [5], [6]].

However, due to the unpredictability and intermittency of the renewable energy sources employed, optimizing and scaling HRES capacity remains a considerable difficulty [[7], [8], [9]]. The difficulty of maximizing and scaling HRES capacity is crucial, since it can have a substantial influence on the system's cost and dependability [10]. As a result, optimization strategies for improving the effectiveness and reliability of HRES have been presented [11]. Further, zero-carbon or low-carbon energy systems can be the promising options towards a sustainable energy economy [12,13].

Gusain et al. [14] compared optimization approaches for developing the optimal capacity of HRES. According to objective functions, decision factors, and assessment metrics, the study divided techniques into conventional, modern, or hybrid. In contrast to earlier studies that highlight technological reliability and economic views, this research emphasizes the significance of environmental indicators. Because of their flexibility and efficiency, hybrid meta-heuristic optimization strategies are becoming increasingly popular, according to the research findings. Although economic metrics are the most widely employed, there is a rising trend toward multi-objective functions that take both economic and reliability considerations into account in the optimum design of HRES.

In this case, Thirunavukkarasu et al. [15] catered a concise overview of the latest advancements in the optimization of different HRES through different approaches to optimization, such as software-based optimization tools artificial intelligence (AI), traditional methods, hybrid algorithms. When compared to traditional approaches, the adoption of AI-based methodologies has demonstrated significant potential and provides a worldwide answer in much less time. Even though, there are certain drawbacks to AI-based approaches.

To address these restrictions, a hybrid optimization technique, which combines more than one algorithm, can be used to solve problems more efficiently and consistently. There are also various popular optimization software programs available, with HOMER being one of the most extensively utilized owing to its simplicity [16]. As HRES optimization studies advance, it is critical to prioritize renewable energy sources [15]. This review seeks to give readers with insights into existing and emerging optimization methodologies for HRES applications, allowing them to select the best strategy for their unique needs.

In order to solve complicated optimization problem effectively, metaheuristic algorithms are utilized.

Krishnakumar et al. [17], for example, suggested a hybrid control approach for improving power system dependability in HRES like hybrid fuel cell systems and PV/wind systems. The suggested strategy minimizes the cost function and improves power system dependability by utilizing the Tunicate Swarm algorithm (TSA). The fundamental goal of this research is to lower costs under diverse situations, such as fuel cell, solar, and wind power. The TSA approach is taught utilizing input characteristics such as the available sources' prior instantaneous energy. To give the appropriate control signal and obtain the target RES power, the TSA control system creates gain variables. The suggested system has been implemented in MATLAB/Simulink and its efficacy is compared to those of current approaches such as NSGA-II, DE, MA-RBFNN, and NSGA-III. The findings show that the suggested method performs better those other comparative approaches with a much more stunning effectiveness rating of 97.83 %.

The use of metaheuristic algorithms to optimize the performance of solar systems had also been published in the literature [18,19]. Kishore et al. [20] used combination of teaching-learning and artificial bee colony-based maximum power point tracking design for photovoltaic (PV) systems under diverse partial shading conditions. They reported that the method could ensure a superior performance than other ones. In addition, opposition-based equilibrium optimizer algorithm was employed in Ref. [21] to reduce oscillations around PV systems’ global maximum peak power. The efficiency for that model method under dynamic partial shading condition was around 86.3–96.2 %.

Pan et al. [22] suggested an approach to reduce the running expenses of HRESs, that provide an optimum operating strategy based on PM-GWO (position-mutation gray wolf optimization). To avoid the model collapsing into a local optimum, GWO was enhanced with a position-mutation technique and an indirect convergent factor to address the issue of complicated and nonlinear components. The simulation results showed that the improved HRES operating strategy efficiently used the new energy rate and decreased prices of electricity while preserving reliability of the system and the high cost-efficiency. Bhimaraju et al. [23] presented the best size of grid-connected solar PV/Wind HRES that includes PSHS (pumped storage hydro-power stations). The main aim was to find the best PV-WT-PSHS combinations to reduce the LCOE (levelized cost of energy). The influence of random intake throughout the rainy season on the size of the HRES is also investigated. The optimization was done with a method which is named SSR (search space reduction) and an enhanced search space reduction (ESSR). The results showed that when the random inflow was taken into account, the cost per unit was much lower. The ESSR algorithm's performance was validated by comparing the findings to other well-known algorithms such as GWO and teaching-learning based optimization. The collected findings show that the ESSR method was preferable since it can attain the lowest importance of the goal function when it is compared to the other algorithms.

From the literatures [24,25], the development of HRESs in remote areas is of great importance and popularity. However, measuring and optimizing the capacity of these systems faces a difficult challenge. Multiple works had been reported in the literature to optimize such systems, all of which aim to achieve an optimal configuration with minimum annual net cost. Therefore, a HRES can compete with other similar systems that can address the lowest net annual cost. Based on this, the significance of providing off-grid electrification to remote areas through HRESs (under a minimum net annual cost) can be highlighted as a crucial case for sustainable growth. This study provides a newly modified metaheuristic algorithm, called HGSA to provide promising results in solving off-grid HRES optimization problem. The HGSA is a revised version of the Golden Search Algorithm, which is a one-dimensional optimization algorithm that searches for the minimum value of a function within a given interval. Herein, the aim of this algorithm is to minimize the amount of net cost which is used annually; to reduce the probability of power supply interruption. In order to assess the effectiveness of the proposed algorithm, a simulation study over a long period (30 years) on a remote area (Zabol, located in southeastern Afghanistan) was conducted. Moreover, the suggested HRES is intended to meet load demand by combining wind energy and hydrogen fuel cell technology. The suggested algorithm's goal is to reduce the chance of power supply failure while minimizing the overall net yearly cost of the system. The overall net yearly system cost comprises the HRES's capital, operating, and maintenance expenditures. The chance of power outage owing to inadequate HRES capacity is represented by the loss of power supply probability. The suggested HGSA performs better than the other comparison algorithms regarding performance. As a result, the first donation of the current study is the development of a practical method for long-term capacity planning and optimization of off-grid HRES. The suggested HGSA can greatly contribute to accomplishing development goals which are constant by lowering the overall net yearly system cost and the chance of energy supply failure. In summary, the objectives and contributions of the current article are:

• Developing a practical method for long-term application planning and optimization of off-grid HRES (based on combining wind energy and hydrogen fuel cell technology);
• Minimizing the net annual cost of a HRES in a remote area to reduce the probability of power supply interruption;
• Utilizing of HGSA for optimization, which is a relatively new technique, can achieve superior results;
• Conducting a simulation study over a long period (30 years) on a remote area (Zabol, located in southeastern Afghanistan) to assess the effectiveness of the proposed algorithm;
• Achieving more promising results than the other comparative algorithms, and therefore, planning a valuable tool for long-term applications and optimization of off-grid HRES to achieving sustainable development goals.

In the next section of the article, the proposed system model including the model schematic and mathematical modeling of the components is presented. The third section is related to the optimization problem and the definition of the objective function. In the fourth section, the definition, algorithm implementation and verification of HGSA are presented. In the fifth part, the results obtained from the simulation are discussed. Finally, the general conclusions of the article are presented in the last section.

## 2 Model of the system

### 2.1 Schematic of model

In the current research HRES (hybrid renewable energy system) is used for the analysis [26]. The suggested HRES is intended to meet load demand by combining wind energy and hydrogen fuel cell technology. The wind turbine (WT) generates energy, which is subsequently utilized to power the load in the system [27]. The WT's surplus electricity is utilized to power the Electrolyzer (EL), which separates water molecules into hydrogen and oxygen. The hydrogen has been stored for later use in the hydrogen tank (H_2_ tank).

When the WT cannot provide the demand for power alone, the hydrogen is sent into FC (fuel cell), where it combines with oxygen existing in the air to create water, electricity, and heat [28]. The fuel cell's power is then transformed to the required voltage and frequency by the inverter (Inv) before being supplied to the load. The system is designed to operate in a seamless manner with the WT and the FC which work with each other to supply the load which is demanded. When the WT is generating adequate electricity to power the load, the hydrogen is not used and the FC is idle. However, when the WT is not generating enough electricity to reach the load demand, the FC starts producing electricity using the hydrogen which is saved in the H_2_ tank.

It is critical to balance energy output and consumption while evaluating the lifetime of the components while designing a hybrid system. WT, H_2_ tank, EL, Inv, and FC have lifetimes of 15, 15, 15, 15, and 10 years, respectively, while the project has a lifespan of 15 years. This implies that each component must be built and managed in such a way that their efficiency and longevity are maximized while meeting load demand.

Energy balancing is one method for improving the architecture of the hybrid system. This entails balancing the energy which is produced by the WT and the energy provided by the fuel cell such that the system functions at optimal efficiency while reducing component wear and tear. This may be accomplished by regulating the WT's power output, the hydrogen storage capacity of the H2 tank, and the fuel cell's power output according to the load requirement.

**Fig. 1:**
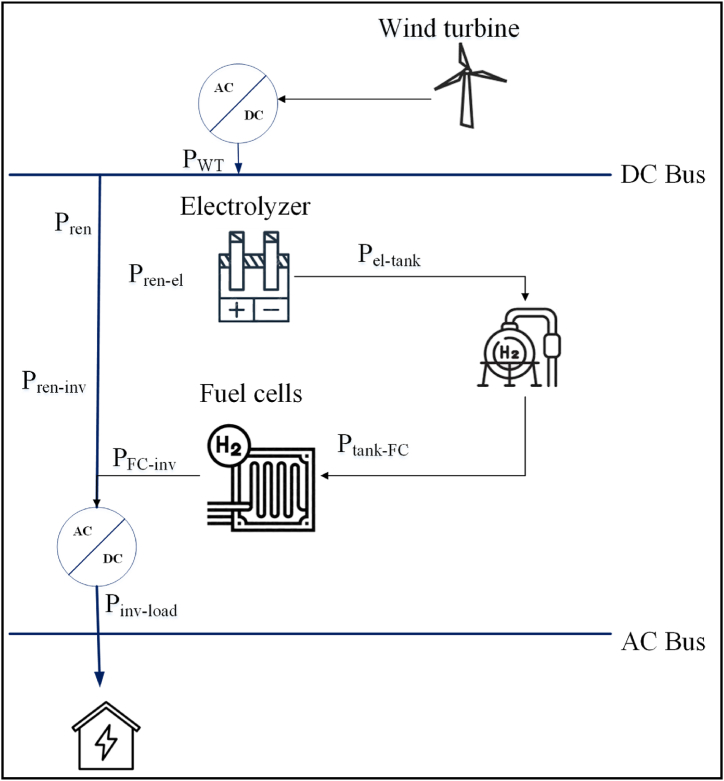
Hybrid system's architecture and its connected components.

At last, constant maintenance and monitoring are essential to guarantee that the hybrid system performs efficiently during its lifespan. This involves monitoring the energy output and consumption of each component, inspecting the components for wear and tear, and replacing components as needed to ensure that the system operates at its optimum performance. Fig. (1) depicts the intended hybrid system's architecture and how each of the components is linked.

The hybrid system is a cost-effective and environmentally friendly energy solution that combines hydrogen fuel cell technology and wind energy to meet load demand. The system can provide a stable and cost-effective supply of electricity while lowering carbon emissions by using diverse energy sources. The diagram depicts how the various system components work together to meet the load requirement.

### 2.2 Mathematical modeling of the components

#### 2.2.1 Wind turbine

WTs are renewable energy technologies that transform the wind kinetic energy into electricity [29]. It consists of a rotor with spinning blades that rotate when the wind blows and a generator that converts rotational energy into electricity [30]. The WTs usually work together with other renewable energy sources in HRES, such as fuel cells, to generate a more reliable and consistent power supply [31]. This is due to the fact that wind turbines rely on wind availability, which varies depending on the weather [32].

The amount of wind energy that a wind turbine can convert into electrical energy is determined by the area which is swept, that pertains to the area covered by the blades in motion. The larger the swept area, the more wind energy can be captured and transformed into electrical energy. The power output which is generated by wind turbine is also influenced by speed of wind and air density. Wind turbines come in various sizes and can generate electricity ranging from a few kilowatts to several megawatts. The power produced by a wind turbine can be calculated using the formula provided below [Equation (1)] [33]:

(1) $$P_{WT}=\left\{ \begin{array}{c} 0\;v<v_{ci}\\ \begin{array}{c} \frac{P_{r}\times \left(v^{3}-v_{cutin}^{3}\right)}{v_{r}^{3}-v_{cutin}^{3}}\;v_{ci}\leq v\leq v_{rated}\;\\ \;{\;P}_{r}\;v_{rated}\leq v\leq v_{co}\\ \;0\;v>v_{co} \end{array} \end{array}\right.$$

where, $v_{rated}$ specifies the rated wind speed, and v, $v_{ci}$, and $v_{co}$, represent, the local, the cut-in, and the cut-out wind speed for the wind turbine generator, respectively. Finally, $P_{r}$ signifies the rated power and is achieved by equation (2):

(2) $$P_{r}=\frac{1}{2}\times C_{p}\times A_{W}\times \rho_{a}\times V_{r}^{3}\times \eta_{r}\times \eta_{w}$$

where, $C_{p}$ specifies the power coefficient, $\rho_{a}$ signifies the air density, $A_{W}$ defines the swept area, and $\eta_{r}$ and $\eta_{W}$ represent, in turn, the reducer efficiency and the WT generator efficiency,

Wind turbine expenses are generally divided into two distinct groups: investment and operation and maintenance. The net annual cost which is invested ($I_{WT\left(NAV\right)}$) is the overall amount spent on the wind turbine, including the original purchase, installation, and any financing charges. This cost is often computed as a net present value and spread out over the life of the wind turbine.

The yearly operation and maintenance cost ($OM_{WT}$) refers to the continuing costs of operating and maintaining the wind turbine, including repairs, maintenance, and monitoring. This cost is likewise spread out throughout the wind turbine's lifetime and varies based on the size and kind of turbine, location, and other considerations.

The total net annual cost (TNAC) of a wind turbine ($TNAC_{WT}$) is the sum of the investment cost and the expense of maintaining and operating throughout the turbine's lifetime. It is given as a single number and is used to assess the wind turbine's economic feasibility. The formula mentioned below may be utilized to ascertain the $TNAC_{WT}$ [Equations (3), (4), (5)]:

(3) $$TNAC_{WT}=I_{WT\left(NAV\right)}+OM_{WT}$$

where,

(4) $$I_{WT\left(NAV\right)}=A_{W}\times C_{WT}\times \left(\frac{i\times {\left(1+i\right)}^{n}}{{\left(1+i\right)}^{n}-1}\right)$$

(5) $$OM_{WT}=A_{W}\times C_{OM-WT}$$

where, $C_{WT}$ and $C_{OM-WT}$ represent, in turn, the unit price of the WTs, which is the sum of the WT cost plus the charge of WT installation, and the yearly operating and the maintenance expense of each WT.

#### 2.2.2 Hydrogen system

According to the search results, a hybrid system comprised of a WT, a H_2_ tank, an EL and a FC is required to maximize the levelized storage cost, as well as the overall yearly expense of system of the wind/PV/H_2_ hybrid [34]. In this hybrid system, the fuel cell provides energy by utilizing hydrogen which is saved in the tank of hydrogen. When wind power is inadequate to satisfy the power requirement, the fuel cell kicks in to assist the wind turbine. In this case, the fuel cell produces energy by using H2 from the hydrogen tank.

##### 2.2.2.1 Fuel cell

A fuel cell's lifespan is determined by various factors, including operating circumstances, fuel quality, and maintenance procedures. Fuel cells have an average lifespan of several years, ranging from 5 to 10 years [35].

A hybrid algorithm which is involved in a bi-level optimum capacity configuration model is provided in a research study to calculate the TNAC of the hybrid system. This model takes seasonal storage characteristics of the hydrogen energy system into account and finds the ideal capacity design of the wind-photovoltaic-hydrogen hybrid system that minimizes the TNAC. The TNAC of FC can be evaluated using the formula below [Equations (6), (7), (8), (9)] [36]:

(6) $$TNAC_{FC}=I_{FC\left(NAV\right)}+OM_{FC}+R_{FC\left(NAV\right)}$$

where,

(7) $$OM_{FC}=N_{FC}\times C_{OM-WT}$$

(8) $$I_{FC\left(NAV\right)}=N_{FC}\times C_{FC}\times \left(\frac{i\times {\left(1+i\right)}^{n}}{{\left(1+i\right)}^{n}-1}\right)$$

(9) $$R_{FC\left(NAV\right)}=R_{FC}\times \left(\sum_{5,7,10}\frac{1}{{\left(1+i\right)}^{n}}\right)\times \left(\frac{i\times {\left(1+i\right)}^{n}}{{\left(1+i\right)}^{n}-1}\right)$$

Where, $R_{FC}$ specifies the replacement cost of a fuel cell, and $C_{FC}$ and $C_{OM-FC}$ represent, in turn, the unit cost of the FCs, and the yearly operating and maintenance cost of each FC.

##### 2.2.2.2 Electrolyzer

When the wind turbine generates more power than needed, an Electrolyzer system can be utilized to convert the excess energy into hydrogen. This process involves splitting water into hydrogen and oxygen using electricity, with the hydrogen being stored for future use. The replacement time for the Electrolyzer is after 15 years of use which is because Electrolyzer has a limited lifespan and may become less efficient or unreliable over time. It is important to replace them accordingly to ensure the continued functioning of the system. TNAC of an Electrolyzer may be calculated in the same way as the TNAC of a hydrogen tank is calculated, which includes the net yearly value of investment cost and maintenance cost and annual operation. The TNAC of an Electrolyzer may be calculated by taking into account the electrolyzer investment expense, maintenance costs and yearly operating, and the cost of energy required to operate the electrolyzer [34].

The Electrolyzer investment cost may be determined using the installation cost, equipment cost, and any other expenditure such as transportation and site preparation. Labor, spare parts and any maintenance or repair services can all be included in the yearly operating and maintenance cost.

The cost of electricity required to run the Electrolyzer can be affected by a number of factors, including the electrolyzer efficacy, the price of power, and the operating circumstances. The cost of electricity may be determined using the electrolyzer power consumption and the price of electricity per kilowatt-hour.

After these expenses have been estimated, the TNAC of the Electrolyzer may be calculated using a formula similar to that of a hydrogen tank [Equations (10), (11), (12), (13)]:

(10) $$TNAC_{El}=I_{EL\left(NAV\right)}+OM_{El}+R_{El\left(NAV\right)}$$

where,

(11) $$OM_{El}=N_{El}\times C_{OM-El}$$

(12) $$I_{El\left(NAV\right)}=N_{El}\times C_{El}\times \left(\frac{i\times {\left(1+i\right)}^{n}}{{\left(1+i\right)}^{n}-1}\right)$$

(13) $$R_{El\left(NAV\right)}=R_{ElC}\times \left(\sum_{5,7,10}\frac{1}{{\left(1+i\right)}^{n}}\right)\times \left(\frac{i\times {\left(1+i\right)}^{n}}{{\left(1+i\right)}^{n}-1}\right)$$

Where, $R_{El}$ specifies the electrolyzer replacement cost, and $C_{El}$ and $C_{OM-El}$ represent, in turn, the unit cost of the electrolyzers, and the yearly operating and maintenance cost of each Electrolyzer.

##### 2.2.2.3 H_2_ tank

The H_2_ can be stored in high-pressure tanks while liquid hydrogen requires cryogenic temperatures. To evaluate TNAC of an electrolyzer, the TNAC of the hydrogen storage tanks is calculated as the net annual value of investment cost and maintenance cost and annual operation. Therefore, the TNAC of the H_2_ storage tank may be calculated using a formula similar to that of a hydrogen tank [Equations (14), (15), (16)]:

(14) $$TNAC_{H2}=I_{H2\left(NAV\right)}+OM_{H2}$$

where,

(15) $$OM_{H2}=N_{H2}\times C_{OM-H2}$$

(16) $$I_{H2\left(NAV\right)}=N_{H2}\times C_{H2}\times \left(\frac{i\times {\left(1+i\right)}^{n}}{{\left(1+i\right)}^{n}-1}\right)$$

where, $N_{H2}$ describes H_2_ storage tanks quantity, and $C_{H2}$ and $C_{OM-H2}$ represent, in turn, the unit cost of the H_2_ storage tanks, and the yearly operating and maintenance cost of each H_2_ storage tank.

#### 2.2.3 Inverter

Inverter in an HRES is used to convert the direct current (DC) electricity generated by solar panels or other renewable sources into alternating current (AC) electricity which can be utilized in homes or buildings. TNAC of an inverter can be evaluated using a cost-benefit analysis [37]. The TNAC of the inverter may be evaluated by a formula similar to that of an inverter [Equations (17), (18), (19), (20)]:

(17) $$TNAC_{inv}=I_{inv\left(NAV\right)}+OM_{inv}+R_{inv\left(NAV\right)}$$

where,

(18) $$OM_{inv}=P_{inv}\times C_{OM-inv}$$

(19) $$I_{inv\left(NAV\right)}=P_{inv}\times C_{inv}\times \left(\frac{i\times {\left(1+i\right)}^{n}}{{\left(1+i\right)}^{n}-1}\right)$$

(20) $$R_{inv\left(NAV\right)}=R_{inv}\times \left(\sum_{15}\frac{1}{{\left(1+i\right)}^{n}}\right)\times \left(\frac{i\times {\left(1+i\right)}^{n}}{{\left(1+i\right)}^{n}-1}\right)$$

where, $P_{inv}$ signifies the rated power of an inverter, and $C_{inv}$ and $C_{OM-inv}$ describe, in turn, the prize of the inverter, and maintenance and the yearly operating cost of the inverters.

### 2.3 Energy management and optimization

Here, a hybrid energy system operation comprising WTs accompanying an ESS (energy storage system) consisting of an Electrolyzer, hydrogen tanks, and fuel cell has been explained. by considering that the value of the absolute power produced by the wind turbines at hour t is equal to the power output of the WTs, $P_{G}=P_{WT}$, and the load demand, $P_{L}$, in a determinative range, the absolute power produced may either surpass or fall below the demand which is expected, with intervals measured in 1-h increments in this particular study.

If $P_{G}\left(t\right)$ is greater than or equal to the $\frac{P_{L}\left(t\right)}{\eta_{inv}}$, the Electrolyzer has been utilized to fill the H_2_ storage tanks. The quantity of the H_2_ storage tanks is represented by HST and is described by the following equation [Equation (21)]:

(21) $$HST\left(t\right)=HST\left(t-1\right)+\left(E_{G}\left(t\right)-\frac{E_{L}\left(t\right)}{\eta_{inv}}\right)\times \eta_{El}$$

where, the load demand has been achieved in kilowatt-hours (kWh) and can be represented by $E_{L}$, the generated energy is defined by $E_{G}$, $\eta_{El}$ describes the Electrolyzer efficiency, $\eta_{inv}$ specifies the efficiency of the inverter, and HST(t) and HST(t−1) represent, inurn, the amount of energy stored in the hydrogen tanks at t and t−1.

As the maximum amount of energy that can be kept in the hydrogen tanks is limited to $HST_{\max}$, the equation below has been considered in the optimization process [Equation (22)]:

(22) $$HST\left(t\right)\geq HST_{\min}$$

when utilizing the fuel cell, while HST(t) is less than $HST_{\min}$, a load ratio has not fulfilled.

Under these circumstances, the HST(t) amount equals $HST_{\min}$, and the LPS (loss of power supply) has been evaluated by equation (23):

(23) $$LPS\left(t\right)=\frac{E_{L}\left(t\right)}{\eta_{inv}}-E_{G}\left(t\right)-\left(HST\left(t-1\right)-HST_{\min}\right)\times \eta_{FC}$$

## 3 Optimization problem

### 3.1 Objective function

In the given method optimization issue, the main purpose of objective function is to minimize TNAC of the hybrid renewable energy system. The TNAC is a comprehensive measure that takes into account the capital costs, maintenance costs of the system, and operating costs over its lifetime. By minimizing the TNAC, the goal is to design a system that provides reliable and sustainable energy while also being cost-effective. This involves finding the optimal balance between the investment in capital equipment and the resulting energy output, as well as optimizing the energy storage and delivery mechanisms to minimize waste and maximize efficiency. The objective function is as follows [Equation (24)]:

(24) $$TNAC{|}_{A_{WT},N_{HT}}=\sum_{HT,WT,El,Inv,FC}I_{m\left(NAV\right)}+OM_{m}+R_{m\left(NAV\right)}$$

in the given design problem, the hybrid renewable energy system consists of various components, such as H_2_ tank, WT, El, FC, and inv. The maximum number of these components is not clear, which presents a challenge in optimizing the system design.

### 3.2 Constraints

The design challenge contains constraints that limit the maximum number of each component to handle this issue. For example, there may be a limit on the number of wind turbines that can be placed in a certain region. Similarly, the highest capacity of the energy storage system, the optimum effectiveness of the energy conversion processes, or the highest level of demand that the system can fulfill may be limited.

The objective is to establish a system design that fulfills the energy demand while also being feasible and practical to deploy by factoring these restrictions into the design challenge. These restrictions contribute to the system's optimization within the confines of available resources and technology.

In general, including restrictions in the design issue is a vital step in creating an efficient and feasible hybrid renewable energy system. The resulting system architecture is more likely to be sustainable and effective in satisfying energy demand if the limits of existing resources and technology are considered. Here are the utilized constraints in this study [Equation (25)]:

(25) $$\left[\begin{array}{c} 0\\ 0 \end{array}\right]\leq \left[\begin{array}{c} A_{W}\\ N_{HT} \end{array}\right]\leq \left[\begin{array}{c} A_{WT}^{\max}\\ N_{HT}^{\max} \end{array}\right]$$

To create a reliable hybrid energy system, we need to consider the term “loss of power supply reliability, β”. The term β is a value between 0 and 1, where 0 means the system reliably meets the energy demand, and 1 means the opposite. We can calculate β over a specific time period (1 year in this research) using the following formula [Equation (26)]:

(26) $$\beta =\frac{\sum_{t=1}^{T}LPS\left(t\right)}{\sum_{t=1}^{T}E_{L}\left(t\right)}$$

Y considering the loss of power supply reliability, the other constraint can be as follows [Equation (27)]:

(27) $$\beta \leq \beta_{D}$$

where, $\beta_{D}$ describes the maximum feasible power supply reliability.

## 4 Hybrid golden search algorithm

When designing a HRES, it's important to determine the appropriate size of the system [38]. Two significant factors to consider when deciding on the system size are reliability and cost. For example, making the system larger can improve its reliability but also increase its cost [39]. One beneficial technique to achieve these decision variables is using metaheuristic algorithms. In recent years, a method called Golden Search Algorithm has been proposed for global optimization problems. In this study, we utilized a hybrid version of this algorithm for optimizing the design of hybrid energy system.

In this section, the authors introduce the proposed hybrid golden search algorithm and the method of its improvement.

Numerous stochastic optimization approaches for finding the optimum solution were devised, which typically entail producing a group of plausible solutions, referred to as the initial population, using a random process. The fitness of these solutions is assessed using a fitness function at each iteration, and they are adjusted using several formulae that constitute the backbone of the optimization approach. The process of iterative modification continues until a sufficient termination condition is fulfilled.

This work offers the Golden Search Optimizer (GSO), a new optimization technique based on bio-inspired algorithms. The GSO combines the advantages of prior approaches like SCA (sine cosine algorithm) and PSO (particle swarm optimization) to give a well-balanced strategy between local exploitation and global exploration, preventing early convergence. The technique updates object positions using step size parameters similar to velocity in PSO, however, sine and cosine functions are used instead of random values. These functions' oscillation features allow one item to travel across another and efficiently utilize the area between two solutions.

Furthermore, the method's exploration capabilities may be expanded by broadening the scope of these functions to change the placement of a solution outside the given space. GSO features an easy-to-implement design and beats earlier bio-inspired techniques regarding convergence to the ideal global solution. The GSO method begins with a random alternative solution, and the state of each participant is regenerated after each iteration while satisfying the end condition using a stage size constraint. The GSO is a global optimization approach that seeks to produce an optimal solution by balancing the exploitation and exploration stages. The technique consists of three primary elements: individual initialization, individual evaluation, and individual renewal. The GSO algorithm is explained in detail below.

- Stage 1: Individuals' initialization

The GSO's search procedure begins by forming a group of candidates inside the search region using a specified algorithm. These people are chosen at random to begin the search process, guaranteeing a varied variety of possible results for the optimization issue. The candidates can be modeled as follows [Equation (28)]:

(28) $$G_{i}=lb_{i}+r\times \left(ub_{i}-lb_{i}\right),i=1,2,\ldots N$$

where $G_{i}$ describes the $i^{th}$ candidate in the solution space, and $lb_{i}$ and $ub_{i}$ include the candidate's lower and higher constraints, respectively.

- Stage 2: Individual evaluation

The value of the individuals' fitness in the population is assessed using an objective function during this stage. The objective function is utilized to judge how good the solution is since it measures how effectively the solution fulfills the issue constraints.

- Stage 3: Existing persons must be updated.

After reviewing their fitness values, the process of upgrading individuals begins. Individuals' positions are updated depending on a step size parameter that generates new solutions using sine and cosine functions. The fitness function is then utilized to assess the new solutions, and the procedure is repeated until the termination condition is reached.

- Stage 4: evaluation of stage magnitude

Using the stage magnitude operator (${\text{St}}_{i}$), the agents are shifted to the best outcome in each repetition of the optimization producer. The St calculation is made up of three elements. The first segment illustrates the prior size of the step increment, which is multiplied by T (transformoperator)and gradually reduces to create ideal balance between the algorithm's global and local search.

In the second portion, the distance between an agent's current placement and its prior best placement is determined using the cosine of a variable value between 0 and 1. The last part shows how distant the $i^{th}$ agent is from the optimal position obtained by all agents so far.

**Fig. 2:**
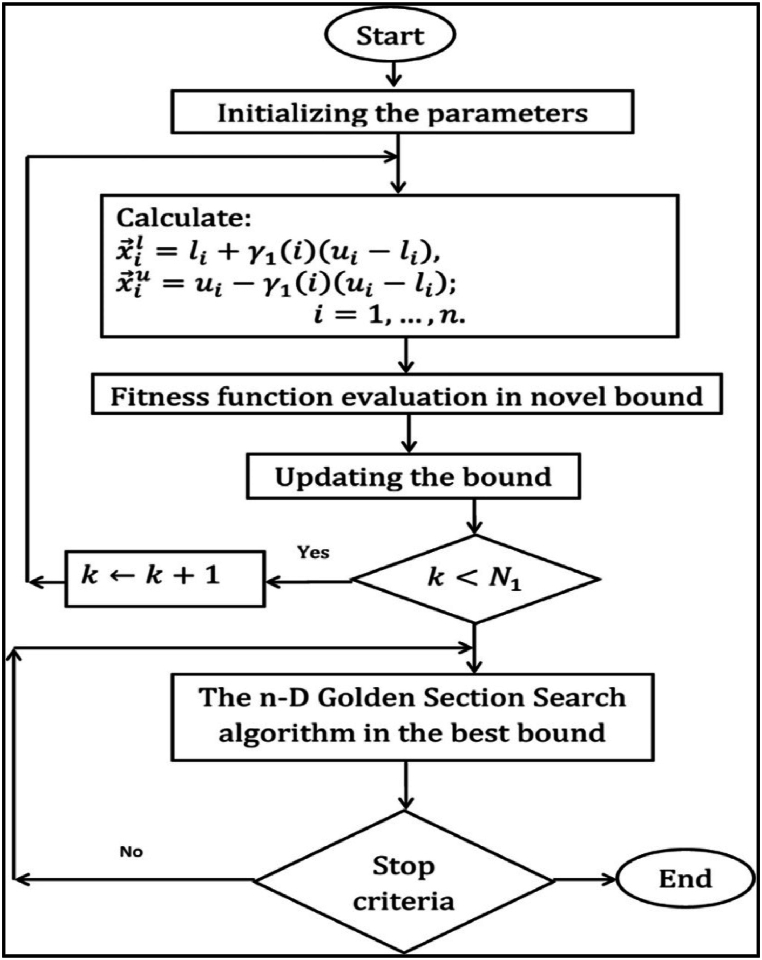
Flowchart of the golden section search optimization algorithm, adopted from [40]. (For interpretation of the references to colour in this figure legend, the reader is referred to the Web version of this article.)

This distance is then multiplied by the sine of a random number that ranges from zero and one. ${\text{St}}_{i}$ will be produced in a random manner during the initial phase of the optimization procedure, and subsequently modified over the iteration's accordance with the given equation [Equation (29)]:

(29) $${St}_{i}\left(t+1\right)=T\times {St}_{i}\left(t\right)+A_{1}\;\cos\left(z_{1}\right)\times \left({Gbest}_{i}-X_{i}\left(t\right)\right)+A_{2}\;\sin\left(z_{2}\right)\times \left({Gbest}_{i}-X_{i}\left(t\right)\right)$$

where, the most optimum location of the $i^{th}$ agent up to the current iteration is represented by ${Gbest}_{i}$. The proposed method involves generating two random numbers, $A_{1}$ and $A_{2}$, within the range of 0–2 and two randomly produced values, $z_{1}$ and r $z_{2}$, within the range of 0–1. To improve search efficiency during exploration and exploitation phases, a transfer operator T is used, which adjusts the search strategy to regulate global and local search. The term T is a function that decays over time and is evaluated as follows [Equation (30)]:

(30) $$T=100\times \exp\left(\frac{-20t}{T_{M}}\;\right)$$

where $T_{M}$ specifies the highest amount of iterations that can be achieved. Fig. 2 depicts the flowchart of the golden section search optimization algorithm, adopted from Ref. [40].

Stage 5: Restriction for stage magnitude

The algorithm adjusts the distance of the agent's movement in all dimensions of the problem space during each iteration. The stage magnitude, a random variable that can cause the agents to move over larger cycles in the problem space, is limited by a specific range to avoid divergence and explosion. This is known as stage magnitude restriction, and it is used to control the agent's movement and prevent them from wandering over broader cycles in the trouble space, ensuring that the algorithm converges to the optimal solution. The preceding equation demonstrates this concept [Equation (31)].

(31) $${St}_{i}\in \left[{-St}_{imin},{St}_{imax}\right]$$

where, ${St}_{imax}$ and ${-St}_{imin}$ represent, in turn, the maximum and the minimum limitations of the movement value with an agent that undergo in its positional coordinates during a single iteration. This limit is defined by equation (32):

(32) $${St}_{imax}=0.1\times \left({ub}_{i}-\;{lb}_{i}\;\right)$$

- Stage 6: renewal situation

In this stage, a new agent is created to explore the global optimum using the following equation, allowing the algorithm to progress towards the optimal solution in the exploration area [Equation (33)]:

(33) $$G_{i}\left(t+1\right)=G_{i}\left(t\right)+{St}_{i}\left(t+1\right)$$

### 4.1 Hybrid golden search algorithm

Although the Golden Search Algorithm has some advantages for tackling optimization issues, it may experience certain difficulties, as every algorithm does. In some difficult situations, the method may have sluggish convergence and produce poor results, despite the fact that it can overcome local minimums. To address these issues, scholars have suggested a number of approaches, including combining the capabilities of other algorithms with the Golden Search Algorithm. To get better outcomes, the current essay offers a hybrid approach that integrates the Golden Search approach with the PSO algorithm. Similarly, to the PSO algorithm, each person in the solution uses their own expertise as well as the knowledge of other candidates to better their next move. This algorithm's two key terms are velocity and position.

In the particle swarm optimization technique, velocity is defined as follows [Equation (34)]:

(34) $$v_{new}=c_{1}\times \left(G_{L}\left(t\right)-G\left(t\right)\right)+c_{2}\times \left(G_{G}\left(t\right)-G\left(t\right)\right)+wv$$

The PSO enhancing equation is represented by its subsequent formula, where $c_{1}$ and $c_{2}$ are the moving factors for the local and global best solutions, $Z_{i}^{G}$ and $Z_{i}^{L}$ have descriptions of the global best position and the current best position for optimization, v and $v_{new}$ are the prior and present particle velocities, and, respectively. A weighting formula is applied with the PSO updating equation to update the particle's location, yielding the following formula [Equation (35)]:

(35) $$G_{i}\left(t+1\right)=G_{i}\left(t\right)+\alpha \times {St}_{i}\left(t+1\right)+\left(1-\alpha \right)\times rand\times v_{new}\times {St}_{i}\left(t+1\right)$$

where, α is set to 0.4 to give additional weight to the PSO-based exploration.

### 4.2 Algorithm verification

Algorithm validation is a vital step in the growth and application of metaheuristic algorithms. By validating the algorithm's performance, researchers can assess its strengths and limitations and determine whether it is appropriate for solving specific optimization issues. Algorithm validation involves testing the algorithm on a set of benchmark problems and evaluating its efficiency based on various metrics, such as solution quality, convergence speed, and computational efficiency. This section presents the results of algorithm validation for the proposed metaheuristic, providing insights into its performance and effectiveness in solving optimization problems. The validation process includes comparisons with other modern algorithms, comprising Pelican Optimization Algorithm (POA) [41], Sine Cosine Algorithm (SCA) [42], Multi-verse optimizer (MVO) [43], Pigeon-inspired Optimization Algorithm (PIOA) [44], to demonstrate the proposed metaheuristic's superiority and potential for real-world applications. Table 1 gives the required parameter setting which is considered for each algorithm.

**Table 1:** The required parameter setting for each algorithm.

Algorithm	Parameter	Value
Pelican Optimization Algorithm (POA) [41]	I	2
	R	0.6
	T	200
Sine Cosine Algorithm (SCA) [42]	Agents for searching	70
	amount of elites	2
	amount of productions	200
Multi-verse optimizer (MVO) [43]	$WEP_{\min}$	0.1
	$WEP_{\max}$	1
	P	5
Pigeon-inspired Optimization Algorithm (PIO) [44]	Number of Pigeons	200
	Dimension of space	15
	Factors of map and compass	0.1
	Operation limit of map and compass	150
	Operation limit of landmark	200
	w	1
	$c_{1}$	1.5
	$c_{2}$	1.5

The main purpose is to lessen the functions. Validating a metaheuristic algorithm based on some benchmark issues that soundly indicate the complexity of real-world optimization issue is crucial in assessing its performance. In the current study, we validate the suggested metaheuristic by using ten test functions from the CEC-BC-2017 test suite (F1, F3, F5, F7, F9, F11, F13, F15, F17, and F19), which is a well-known benchmark for evaluating optimization algorithms' performance. These test functions are designed to represent a range of complex optimization problems and are commonly utilized to assess the efficiency of metaheuristic algorithms. The proposed Hybrid Golden Search Algorithm's effectiveness and scalability to solve real-world optimization issues are assessed by validating it on these test functions. The constraint for all decision variables are (−100, 100).

Validation of a metaheuristic algorithm is crucial to demonstrate its effectiveness and superiority over other modern algorithms. One way to present the results of the algorithm validation is by using a table that includes the average value and standard deviation of the error when contrasted to other algorithms. This table allows researchers to contrast the suggested metaheuristic's efficiency with other algorithms and assess its effectiveness in solving optimization problems.

The average value of the error provides an overall measure of the algorithm's performance, while the standard deviation value represents the variation in the algorithm's performance across different test functions. By presenting the validation results in a table format, researchers can easily compare and analyze the metaheuristic's performance and draw conclusions about its potential for real-world applications. Table 2 indicates the comparison results.

**Table 2:** Comparison results of the suggested HGSA method on the CEC-BC-2017 test suite.

Test function		HGSA	POA	SCA	MVO	PIO
F1	AVG	0.00	4.69	4.43	3.71	3.45
	STD	0.00	3.97	4.14	3.37	2.99
F2	AVG	0.13	4.98	4.51	4.28	4.18
	STD	0.05	4.47	4.23	3.99	3.52
F3	AVG	0.00	5.71e-3	7.89e-3	4.78e-4	6.95e-4
	STD	0.00	4.85e-4	5.43e-4	6.51e-5	8.68e-5
F4	AVG	0.00	0.00	4.41e-6	3.17e-6	2.95e-7
	STD	0.00	0.00	6.84e-6	8.95e-6	4.53e-7
F5	AVG	0.00	3.18	2.23	1.69	1.47
	STD	0.00	2.85	2.04	1.44	1.28
F6	AVG	0.03	1.97	1.84	1.74	1.50
	STD	0.01	1.74	1.61	1.43	1.36
F7	AVG	0.04	5.59	5.42	5.26	4.19
	STD	0.02	5.13	5.01	4.84	3.96
F8	AVG	0.00	1.26	0.91	0.87	0.95
	STD	0.00	1.11	0.83	0.72	0.62
F9	AVG	0.00	0.00	1.32	1.25	1.12
	STD	0.00	0.00	1.24	1.15	0.93
F10	AVG	0.00	2.25	1.99	1.56	1.19
	STD	0.00	1.74	1.48	1.25	1.08

The efficiency of the recommended HGSA in solving the CEC benchmark functions was evaluated and compared with other approaches, and the results are presented in Table 2. The analysis indicates that the HGSA achieved the highest accuracy among all the compared methods, indicating its superior efficiency in solving optimization problems. Moreover, the standard deviation (STD) value of the HGSA was found to be one of the lowest among the compared methods, which suggests that it has a higher reliability in solving optimization problems over multiple runs. The smaller standard deviation value of the HGSA compared to other methods highlights its ability to produce consistent and accurate results during different runs, indicating its higher reliability.

## 5 Simulations and results

The current study proposes a new optimization algorithm, which is named the Hybrid Golden Search Algorithm (HGSA) for finding the optimal number of elements in a hybrid system, subject to predefined constraints. The hybrid system considered in the current study is designed to meet the energy demands of a specific location, which in this case is Zabol in Afghanistan. The main goal of this research is to ascertain the optimal configuration of the hybrid system that can prepare reliable and cost-effective energy to the people of Zabol.

Zabol, located in southeastern Afghanistan, has a moderate to high potential for wind energy due to its geographical location and topography. The city is situated in a mountainous region with significant variations in elevation, which creates ideal wind conditions for electricity generation. According to the recent wind resource assessment implemented by the National Renewable Energy Laboratory, Zabol has an average annual wind speed of 5.5 m per second (m/s) at a height of 50 m, which is considered a good wind resource for electricity generation [45].

The wind speed varies throughout the year, with higher wind speeds occurring during the winter months. Based on the wind resource assessment, it has been estimated that Zabol has the potential to generate between 500 and 800 kW (kW) of electricity per wind turbine, depending on the specific design and location of the turbine. This suggests that wind energy can be a feasible element for meeting the energy needs of Zabol, particularly in conjunction with other renewable energy sources such as solar power.

The Hybrid Golden Search Algorithm combines the strengths of the Golden Section Search and the Particle Swarm Optimization algorithms to achieve a better balance between exploitation and investigation of the space of search. The suggested algorithm is applied to the case study of Zabol to determine the optimal quantity of each component in the hybrid system, including the solar panels, wind turbines, and diesel generators, while adhering to various constraints such as the maximum allowable capital cost and the minimum required reliability level. The results obtained from the application of the Hybrid Golden Search Algorithm show that the suggested algorithm is able to find the optimal configuration of the hybrid system in a relatively short time. The optimal configuration obtained is a trade-off between the cost and reliability of the system, and it provides a cost-effective and reliable energy solution for Zabol.

In the case study, the project lifetime and interest rate were set to 30 years and 15 %, respectively. These parameters were chosen based on various factors such as the expected return on investment, the financial feasibility of the project, and the expected lifespan of the energy system. The interest rate of 15 % indicates the cost of borrowing money or the return on investment required to compensate for the risk associated with the project. This rate is commonly used in energy project financing and is considered typical for projects in developing countries such as Afghanistan.

The project lifetime of 30 years represents the expected lifespan of the energy system. This is an important parameter as it affects the overall cost of the project and its long-term sustainability. A longer project lifetime allows for a greater return on investment and can help ensure the financial viability of the project over its lifespan. Table 3 presents the system components utilized in the current study.

**Table 3:** Component vales used in the case study.

Parameters of wind turbine		Parameters of fuel cell		Parameters of electrolyser	
CMnt−WT	120 $/year	Nominal FC power	4 kw	Nominal EI power	4 kw
Vr	10 m/s	C_OM-FC_	189 $/year	C_OM-EI_	38 $/year
Vco	22 m/s	C_FC_	10000 $	R_EL_	5000 $
Vci	4 m/s	R_FC_	8000 $	C_EI_	7000 $
CWT	1943 $	ηFC	60 %	ηFC	76 %
PT-WT	1 kw	**Parameters of hydrogen tank**		**Parameters of inverter**	
C_p_	0.62	CHT	3000 $	C_INV_	1695 $
ηWT	87 %	COM-HT	45 $/year	P_INV_	4 kw
ρa	1.337 $kg/m^{3}$	Nominal capacity of HT	0.4 kwh	$\eta_{INV}$	96 %

This table includes various parameters such as the load demand, speed of wind, solar irradiation, and the technical specifications of the energy system components such as the solar panels, wind turbines, and batteries. These parameters were utilized to model the energy system and determine the optimal configuration using the Hybrid Golden Search Algorithm. MATLAB R2018b environment is utilized to solve the problem of design optimization.

In the current study, the Hybrid Golden Search Algorithm (HGSA) was used with varied highest allowable power supply reliability loss ($\beta_{D}$) values of 0.01, 0.02, and 0.03. The performance of HGSA was contrasted to other modern algorithms, including Fuzzy Logic and Optimization (Fuzzy-OP) [48], ABC optimization method [49], and the original GSA. The results of the different algorithms were evaluated and compared to determine their effectiveness in solving the optimization problem.

Specifically, each algorithm was administered 30 times to ensure that the results were consistent and not just a result of chance or randomness. To further compare the efficiency of the most incredible algorithms, the maximum number of iterations (maximum iteration) was varied. The algorithms were run with maximum iteration values of 800 over 30 independent runs. By running the algorithms with different maximum iteration values and multiple independent runs, the researchers could assess the efficiency of the algorithms under various conditions and obtain a more comprehensive understanding of their effectiveness. Table 4 presents a comparison of the proposed method and other techniques that were studied in relation to various levels of reliability (ranging from 1 % to 3 %) for a hybrid energy system. The table provides information on how the different techniques performed under varied reliability level for the hybrid scheme.

**Table 4:** Comparison of the suggested approach and other techniques in relation to various levels of reliability for a hybrid energy system.

Level/index of reliability	Mean. ($)	STD ($)	Total Time (sec)
$\beta_{D}=0.1$			
ABC [49]	34,269,700	3,236,280	321.32
Fuzzy-OP [48]	33,628,170	2,568,210	352.32
GSA	34,856,870	4,821,560	326.89
HGSA	32,896,240	2,289,240	369.75
$\beta_{D}=0.02$			
ABC [49]	32,826,730	2,584,210	361.52
Fuzzy-OP [48]	34,583,850	2,454,940	392.47
GSA	35,369,740	5,462,420	316.89
HGSA	32560,740	2,538,520	421.87
$\beta_{D}=0.03$			
ABC [49]	31,542,230	2,750,510	312.63
Fuzzy-OP [48]	31,134,250	2,376,110	398.25
GSA	33,567,730	5,354,420	341.75
HGSA	31,539,810	2,327,260	369.85

Table 4 demonstrates that HGSA outperforms Fuzzy-OP, ABC, and GSA techniques at a reliability level of 1 % ($\beta_{D}=1\%$). The ABC technique shows better performance than the other techniques regarding the index of Total Time, with a time of 321.32 s. Additionally, the HGSA technique obtains the lowest value of fitness function for the hybrid system of 32,896,240$.

At a reliability level of 2 % ($\beta_{D}=2\%$), the HGSA technique obtains the lowest value of fitness function for the hybrid system, which is 32560,740 $. This result shows that the efficiency of outperforms Fuzzy-OP, ABC, and GSA techniques. Fig. (4) presents the minimal value of fitness function of the hybrid system gained by varied algorithms at different levels of reliability (ranging from 1 % to 3 %) for the first 800 iterations. The figure demonstrates that at a reliability level of 2 %, the HGSA technique obtains the lowest fitness function value, indicating its superior performance compared to the other techniques. Additionally, the figure shows that the performance of the HGSA technique remains consistently better than the other techniques across varied reliability levels for the first 800 iterations.

The HGSA technique obtains the lowest value of fitness function for the hybrid system at a reliability level of 3 % ($\beta_{D}=3\%$), which is 31,539,810$. This result demonstrates that the efficiency of HGSA outperforms Fuzzy-OP, ABC, and GSA techniques.

**Fig. 3:**
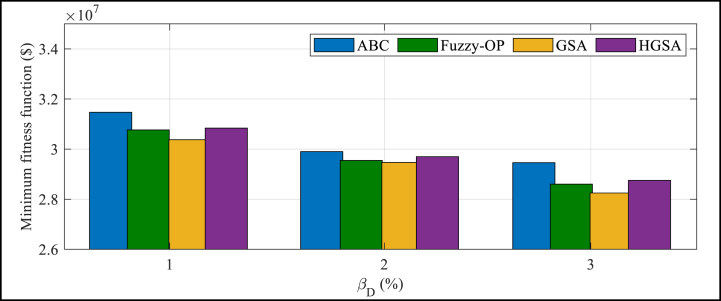
Minimal value of fitness function of the hybrid system gained by varied algorithms at various levels of reliability.

Fig. (3) displays the minimal value of fitness function of the hybrid system gained by varied algorithms at various levels of reliability (ranging from 1 % to 3 %) for the first 800 iterations.

**Fig. 4:**
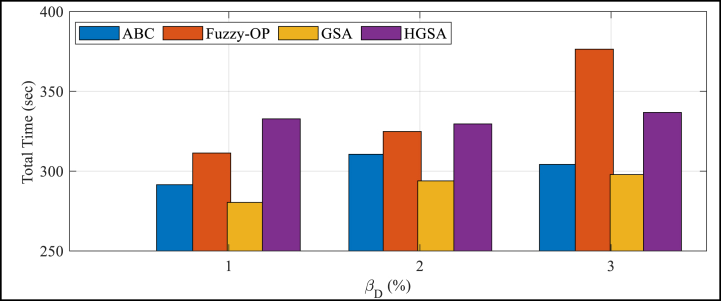
Best simulation time for the comparative algorithms for optimal problem measurment

The figure illustrates that at a reliability level of 3 %, HGSA technique achieves the lowest fitness function value, indicating its superior performance compared to the other techniques. Additionally, the figure shows that the performance of the HGSA technique remains consistently better than the other techniques across varied reliability levels for the first 800 iterations. Fig. (4) illustrates the most amazing simulation time of HGSA, Fuzzy-OP, ABC, and GSA techniques in varied levels of reliability to find out how great the problem is at first 800 iterations.

Based on the evaluation of various levels of reliability, HGSA outperforms the other investigated algorithms in terms of performance. The statistical analysis indicates that the ranking of the algorithms, based on their performance in different reliability levels, is HGSA, Fuzzy-OP, ABC, and GSA techniques. Furthermore, taking the absolute index of simulation time, HGSA demonstrates superior performance compared to Fuzzy-OP, ABC, and GSA techniques. Therefore, it may be inferred that HGSA not only offers sound results than the other explored algorithms but also operates at a high speed. Additionally, the study reveals that the three developed algorithms exhibit better performance than the original algorithm.

The impact of the wind turbine area, $\beta_{D}$ value on the TNAC, and quantity of storage tanks, and is a matter of interest. The suggested algorithms have resolved this problem by solving the problem with varying desired reliability values such as 0.01, 0.02, and 0.03. By setting maximum iteration at 900, Table 5 displays the optimized decision variables attained by the algorithms under different $\beta_{D}$ values.

**Table 5:** Optimized decision variables attained by the algorithms under different $\beta_{D}$ values.

Index	$A_{WT}\left(m^{2}\right)$	$N_{HT}$	TNAC ($)	β%
$\beta_{D}=0.01$				
ABC [49]	224.8	13489	31,825,630	0.9429
Fuzzy-OP [48]	228.9	113,796	31,293,240	0.8561
GSA	234.8	109,689	30,921,360	0.984
HGSA	224.8	131,367	31,446,850	0.9637
$\beta_{D}=0.02$				
ABC [49]	236.7	107,946	30,25,960	1.6359
Fuzzy-OP [48]	2431.8	105,829	29,925,620	2.1236
GSA	213.8	105,744	29,845,850	2.1257
HGSA	234.7	106,668	30,125,870	1.7863
$\beta_{D}=0.03$				
ABC [49]	216.48	105,163	29,78,689	2.8678
Fuzzy-OP [48]	219.8	110,841	28,896,750	2.9324
GSA	213.6	100.152	28,558,40	2.9578
HGSA	211.5	103,863	29,152,630	2.8963

The study reveals that the best algorithm (HGSA) achieves optimal values of 131,367 storage tanks, 224.8 m^2^ wind turbine area, and 0.9637 % when $\beta_{D}$ is set to 1 %. Notably, increasing the $\beta_{D}$ level from 1 % to 2 % causes a decrease in TNAC value from 31,446,850$ to 30,125,870$ in HGSA and also results in a reduction in component size.

When the $\beta_{D}$ value experiences an increase from 2 % to 3 %, wind turbine area, the number of storage tanks, and TNAC values will undergo a decrease of approximately, in turn, 23.2 $m^{2}$, 4886, and3.45 %. As a consequence, the β is increased by 1.11 % ratio.

On the other hand, reducing the $\beta_{D}$ value from 3 % to 1 % will cause the quantity of storage tanks and area of wind turbines to increase by approximately 27,504 and 13.3 $m^{2}$, respectively. Meanwhile, the value of β will decrease by 66.72 %, and TNAC will increase by 7.29 %. It is possible to conduct the same analysis for all algorithms.

To enhance comprehension concerning the impact of the maximum iteration value generated by the HGSA across 30 independent runs, the current study has employed varying maximum iteration values (100, 200, 400, and 800) at different levels of reliability (0%–5%). The findings of this investigation are presented in Table 6.

**Table 6:** Findings of this investigation.

Reliability level	$Iter_{\max}$ value	Mean ($)	STD ($)	Total Time (sec)
$\beta_{D}=0$	100	36,846,680	3,892,120	212.91
	200	36,163,860	3,491,670	293.48
	400	35,927,840	3,482,070	412.66
	800	35,794,870	4,638,980	548.72
$\beta_{D}=0.01$	100	36,157,140	5,139,360	220.86
	200	34,846,710	3,864,780	289.37
	400	33,825,140	3,155,690	402.36
	800	33,445,360	2,918,930	546.14
$\beta_{D}=0.02$	100	35,719,870	5,364,680	241.16
	200	35,368,740	5,336,440	293.54
	400	33,853,560	3,256,350	462.78
	800	31,992,860	2,158,970	537.26
$\beta_{D}=0.05$	100	30.486,230	4,789,340	223.38
	200	30,384,450	3,853,480	293.52
	400	30,138,380	4,998,890	421.65
	800	28,899,490	3,625,760	548.28

The results presented in Table 6 show the impact of the maximum iteration value generated by the HGSA on the optimization performance across 30 independent runs at different levels of reliability. The reliability level is represented by $\beta_{D}$, which ranges from 0 % to 5 %. The four different maximum iteration values tested in this study are 100, 200, 400, and 800. The findings indicate that as the value of β_D increases, the mean total cost decreases, indicating a reduced risk of failure. At a reliability level of 0 %, the mean total cost is the highest for all maximum iteration values. As the maximum iteration value increases, the mean total cost decreases. However, at a reliability level of 0 %, the decrease in the mean total cost is not significant as the maximum iteration value increases. The STD of the mean total expense is also presented in Table 6. The STD decreases as the maximum iteration value increases, indicating a more stable convergence towards the optimal solution. At a reliability level of 0 %, the STD is the highest for all maximum iteration values. The absolute time which is taken for the process of optimization is also presented in Table 6. As the maximum iteration value increases, the absolute time which is taken for the process of optimization also increases. At a reliability level of 0 %, the absolute time which is taken for the process of optimization is the highest for all maximum iteration values.

## 6 Conclusions

To achieve the measuring and optimizing the capacity of the HRESs, a new model was introduced in this article. The study proposed a modified metaheuristic approach, known as the HGSA, for long-term application planning and optimization of the off-grid HRESs. The aim of this algorithm was to minimize the amount of net cost which was used annually; to reduce the probability of power supply interruption. In order to assess the effectiveness of the proposed algorithm, a simulation study over a long period (30 years) on a remote area (Zabol, located in southeastern Afghanistan) was conducted. However, by changing geographical conditions and wind speed data, the proposed system and the obtained results can be expanded to other regions. The primary purpose of the algorithm was to minimize the absolute annual system cost of net while simultaneously reducing the probability of power supply loss. From the results, at a reliability level of 0 %, the mean total cost is the highest for all maximum iteration values. As the maximum iteration value increases, the mean total cost decreases. However, at a reliability level of 0 %, the decrease in the mean total cost is not significant as the maximum iteration value increases. In addition, increasing the reliability level from 1 % to 3 % causes a decrease in the total net annual cost by around 7.3 % under the proposed HGSA and also results in a reduction in component size (around 6 % and 21 % reductions for the wind turbine area and storage tanks, respectively). Further, the HGSA technique obtains the lowest value of fitness function for the hybrid system at a reliability level of 3 %, which is 31,539,810$.

This result demonstrates that the efficiency of HGSA outperforms Fuzzy Logic and Optimization, Artificial Bee Colony (ABC), and GSA techniques. Based on this, the proposed HGSA could lead to more promising results than the other comparative algorithms. Hence, the proposed HGSA can be a valuable tool for long-term application planning and optimization of off-grid HRES, which can contribute significantly to achieving sustainable development goals. The proposed algorithm can help in providing reliable and cost-effective power supply to remote areas, thereby improving the living standards of the population and promoting economic growth. It is recommended to compare the results of the proposed algorithm with other similar algorithms in future works. In addition, applying the proposed algorithm to optimize other HRESs with different configurations and comparing the results can be a comprehensive guide for energy engineers.

## Data availability statement

Research data are not shared.

## CRediT authorship contribution statement

**Gengqiang Huang:** Formal analysis, Data curation, Conceptualization. **Jie Gan:** Formal analysis, Data curation, Conceptualization. **Ying Huang:** Formal analysis, Data curation, Conceptualization. **Homayoun Ebrahimian:** Formal analysis, Data curation, Conceptualization.

## Declaration of competing interest

The authors declare that they have no known competing financial interests or personal relationships that could have appeared to influence the work reported in this paper.

## Figures and Tables

**Fig. 5:**
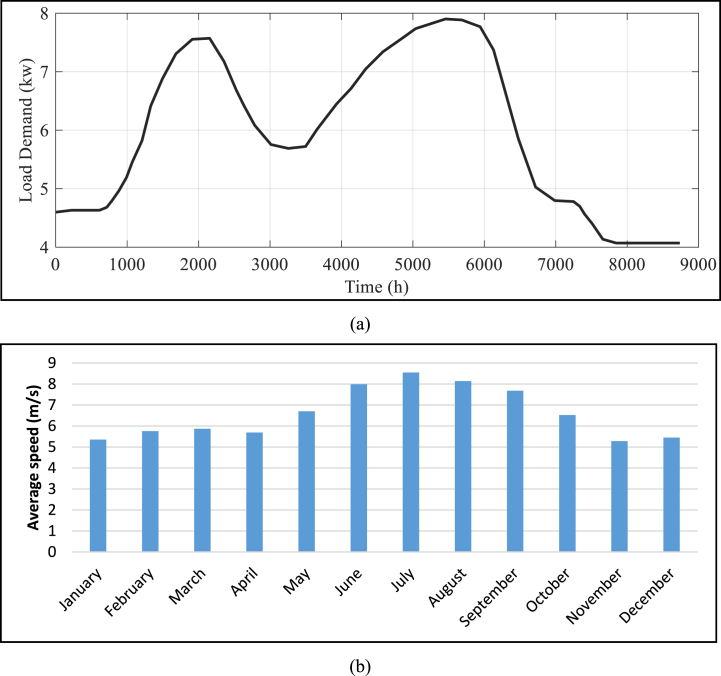
(a) Load profile and (b) wind speed of Zabol region [46,47].

## Appendix

The wind speed and load profile of the area are presented in Fig. (5a) and Fig. (5b).

## References

[1] Zhang G., Xiao C., Razmjooy N. (2022). Optimal operational strategy of hybrid PV/wind renewable energy system using homer: a case study. Int. J. Ambient Energy.

[2] Zhu Ligui (2023). Multi-criteria evaluation and optimization of a novel thermodynamic cycle based on a wind farm, Kalina cycle and storage system: an effort to improve efficiency and sustainability. Sustain. Cities Soc..

[3] Ayadi B. (2024). Multi-criteria/comparative analysis and multi-objective optimization of a hybrid solar/geothermal source system integrated with a carnot battery. Case Stud. Therm. Eng..

[4] Cai W. (2019). Optimal bidding and offering strategies of compressed air energy storage: a hybrid robust-stochastic approach. Renew. Energy.

[5] Ghorbannejad F. (2022). A comprehensive exergoenvironment-emergoeconomic-emergoenvironment based analysis of hybrid steam biomass gasification and solid oxide fuel cell system for a multigeneration system. Energy Rep..

[6] Hasanzadeh A. (2022). Soft computing investigation of stand-alone gas turbine and hybrid gas turbine–solid oxide fuel cell systems via artificial intelligence and multi-objective grey wolf optimizer. Energy Rep..

[7] Chen L. (2022). Optimal modeling of combined cooling, heating, and power systems using developed African Vulture Optimization: a case study in watersport complex. Energy Sources, Part A Recovery, Util. Environ. Eff..

[8] Chen W. (2021). A new design of evolutionary hybrid optimization of SVR model in predicting the blast-induced ground vibration. Eng. Comput..

[9] Zhu W., Nikafshan Rad H., Hasanipanah M. (2021). A chaos recurrent ANFIS optimized by PSO to predict ground vibration generated in rock blasting. Appl. Soft Comput..

[10] Chang Le, Wu Zhixin, Ghadimi Noradin (2023). A new biomass-based hybrid energy system integrated with a flue gas condensation process and energy storage option: an effort to mitigate environmental hazards. Process Saf. Environ. Protect..

[11] Ebrahimian H. (2018). The price prediction for the energy market based on a new method. Economic research-Ekonomska istraživanja.

[12] Luo J. (2024). The optimization of carbon emission prediction in low carbon energy economy under Big data. IEEE Access.

[13] Duan Y., Zhao Y., Hu J. (2023). An initialization-free distributed algorithm for dynamic economic dispatch problems in microgrid: modeling, optimization and analysis. Sustainable Energy, Grids and Networks.

[14] Gusain C., Tripathi M.M., Nangia U. (2023). Study of meta-heuristic optimization methodologies for design of hybrid renewable energy systems. Therm. Sci. Eng. Prog..

[15] Thirunavukkarasu M., Sawle Y., Lala H. (2023). A comprehensive review on optimization of hybrid renewable energy systems using various optimization techniques. Renew. Sustain. Energy Rev..

[16] Jiang Z., Xu C. (2023). Policy incentives, government subsidies, and technological innovation in new energy vehicle enterprises: Evidence from China. Energy Pol..

[17] Krishnakumar R., Ravichandran C. (2022). Reliability and cost minimization of renewable power system with tunicate swarm optimization approach based on the design of PV/Wind/FC system. Renewable Energy Focus.

[18] Kishore D.J.K. (2024). A new metaheuristic-based MPPT controller for photovoltaic systems under partial shading conditions and complex partial shading conditions. Neural Comput. Appl..

[19] Peddakapu K. (2022). A state-of-the-art review on modern and future developments of AGC/LFC of conventional and renewable energy-based power systems. Renewable Energy Focus.

[20] Kishore D.J.K. (2023). Swarm intelligence-based MPPT design for PV systems under diverse partial shading conditions. Energy.

[21] Kishore D.J.K. (2023). A new meta-heuristic optimization-based MPPT control technique for green energy harvesting from photovoltaic systems under different atmospheric conditions. Environ. Sci. Pollut. Control Ser..

[22] Pan T. (2023). Operating strategy for grid-connected solar-wind-battery hybrid systems using improved grey wolf optimization. Elec. Power Syst. Res..

[23] Bhimaraju A., Mahesh A., Joshi S.N. (2022). Techno-economic optimization of grid-connected solar-wind-pumped storage hybrid energy system using improved search space reduction algorithm. J. Energy Storage.

[24] Koholé Y.W. (2024). An effective sizing and sensitivity analysis of a hybrid renewable energy system for household, multi-media and rural healthcare centres power supply: a case study of Kaele, Cameroon. Int. J. Hydrogen Energy.

[25] Oyewole O.L., Nwulu N.I., Okampo E.J. (2024). Optimal design of hydrogen-based storage with a hybrid renewable energy system considering economic and environmental uncertainties. Energy Convers. Manag..

[26] Ghiasi M., et al., Evolution of smart grids towards the Internet of energy: concept and essential components for deep decarbonisation, IET Smart Grid (2022).Volume6, Issue1.

[27] Han E., Ghadimi N. (2022). Model identification of proton-exchange membrane fuel cells based on a hybrid convolutional neural network and extreme learning machine optimized by improved honey badger algorithm. Sustain. Energy Technol. Assessments.

[28] Jiang W. (2022). Optimal economic scheduling of microgrids considering renewable energy sources based on energy hub model using demand response and improved water wave optimization algorithm. J. Energy Storage.

[29] Mehrpooya M. (2021). Numerical investigation of a new combined energy system includes parabolic dish solar collector, Stirling engine and thermoelectric device. Int. J. Energy Res..

[30] Zhang Hua (2024). Efficient design of energy microgrid management system: a promoted Remora optimization algorithm-based approach. Heliyon.

[31] Fan X. (2020). Multi-objective optimization for the proper selection of the best heat pump technology in a fuel cell-heat pump micro-CHP system. Energy Rep..

[32] Zhang Min (2024). Improved chaos grasshopper optimizer and its application to HRES techno-economic evaluation. Heliyon.

[33] Gipe P., Möllerström E. (2023). An overview of the history of wind turbine development: Part II–The 1970s onward. Wind Eng..

[34] Li J. (2023). Optimal sizing for a wind-photovoltaic-hydrogen hybrid system considering levelized cost of storage and source-load interaction. Int. J. Hydrogen Energy.

[35] Arsalis A., Papanastasiou P., Georghiou G.E. (2022). A comparative review of lithium-ion battery and regenerative hydrogen fuel cell technologies for integration with photovoltaic applications. Renew. Energy..

[36] Zhang W. (2021). An artificial intelligence approach to optimization of an off-grid hybrid wind/hydrogen system. Int. J. Hydrogen Energy.

[37] Team L. (2023). Efficiency of inverter: calculation & equation guide. https://www.linquip.com/blog/efficiency-of-inverter/.

[38] Sun L. (2021). Exergy analysis of a fuel cell power system and optimizing it with Fractional-order Coyote Optimization Algorithm. Energy Rep..

[39] Li Shunlei (2024). Evaluating the efficiency of CCHP systems in Xinjiang Uygur Autonomous Region: an optimal strategy based on improved mother optimization algorithm. Case Stud. Therm. Eng..

[40] Koupaei J.A., Hosseini S.M.M., Ghaini F.M.M. (2016). A new optimization algorithm based on chaotic maps and golden section search method. Eng. Appl. Artif. Intell..

[41] Trojovský P., Dehghani M. (2022). Pelican optimization algorithm: a novel nature-inspired algorithm for engineering applications. Sensors.

[42] Mirjalili S.M. (2020). Sine cosine algorithm: theory, literature review, and application in designing bend photonic crystal waveguides. Nature-inspired optimizers: theories, literature reviews and applications.

[43] Mirjalili S., Mirjalili S.M., Hatamlou A. (2016). Multi-verse optimizer: a nature-inspired algorithm for global optimization. Neural Comput. Appl..

[44] Cui Z. (2019). A pigeon-inspired optimization algorithm for many-objective optimization problems. Sci. China Inf. Sci..

[45] Lee N. (2019).

[46] Solargis (2023). Global solar atlas. https://globalsolaratlas.info.

[47] (2020). Weather.

[48] Afolabi T., Farzaneh H. (2023). Optimal design and operation of an off-grid hybrid renewable energy system in Nigeria's rural Residential area, using Fuzzy Logic and optimization techniques. Sustainability.

[49] Wang R., Zhang R. (2023). Techno-economic analysis and optimization of hybrid energy systems based on hydrogen storage for sustainable energy utilization by a biological-inspired optimization algorithm. J. Energy Storage.

